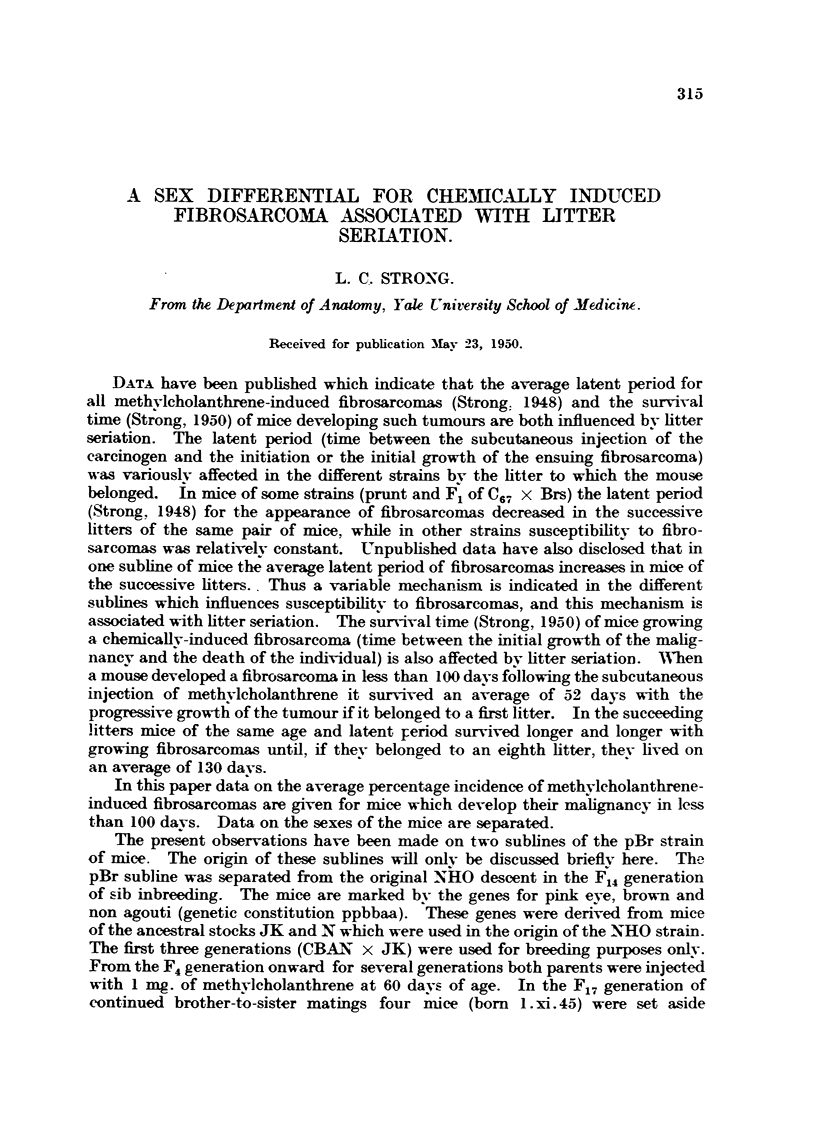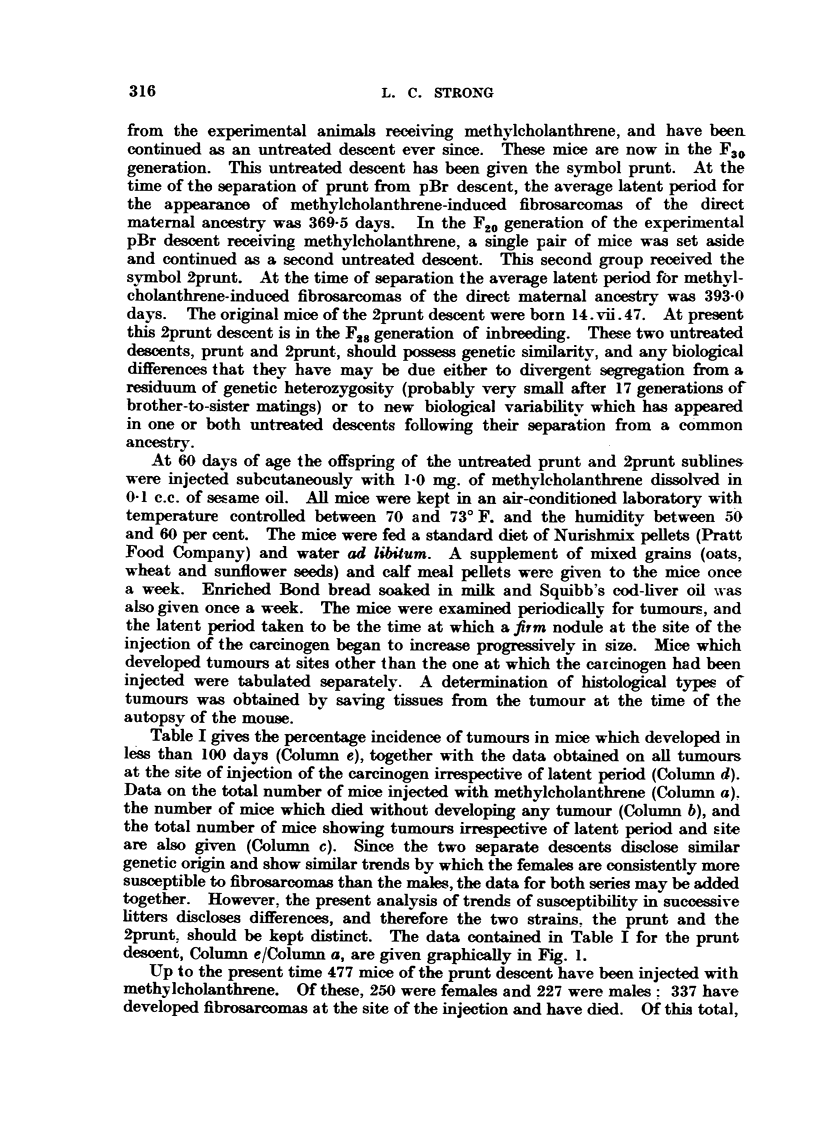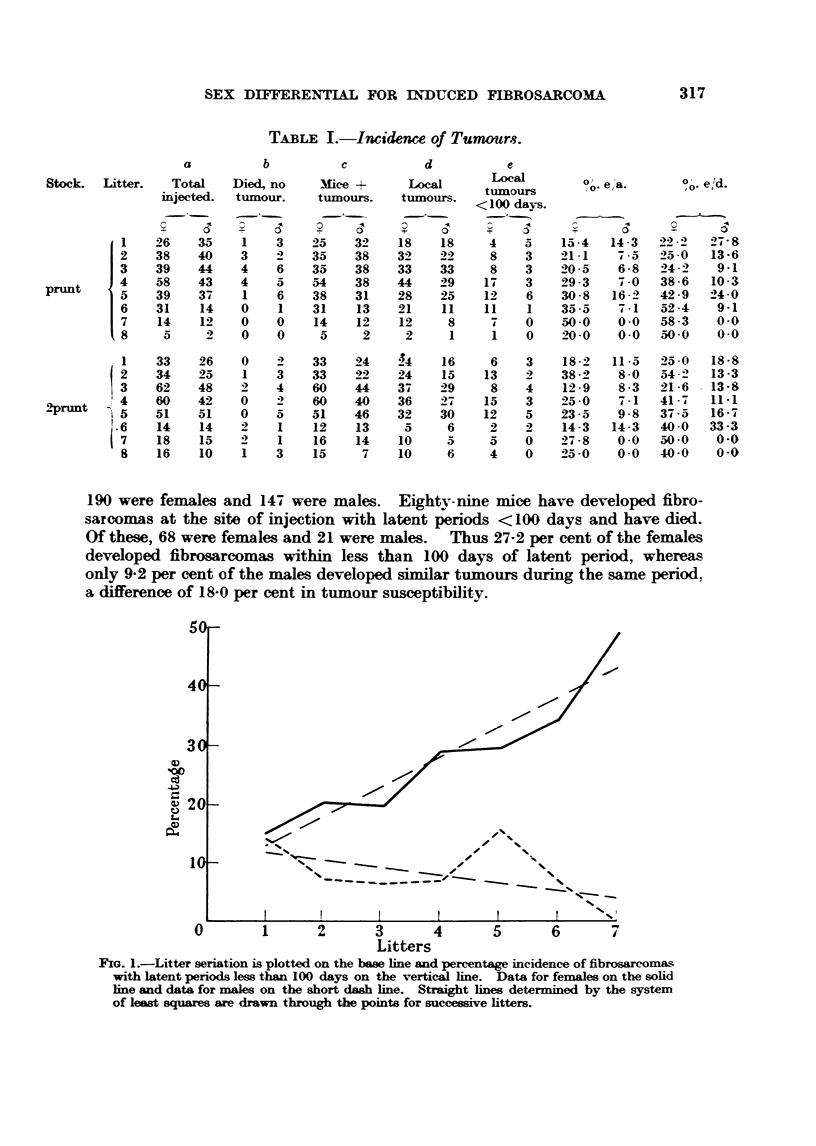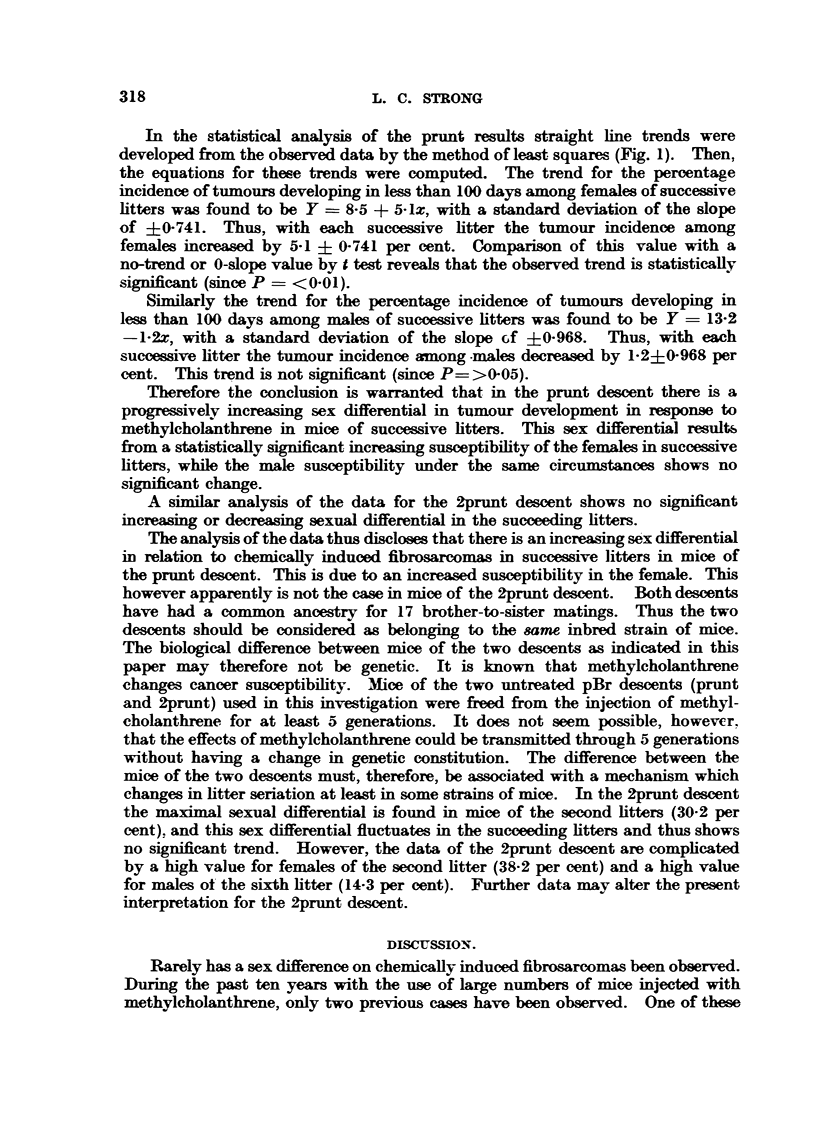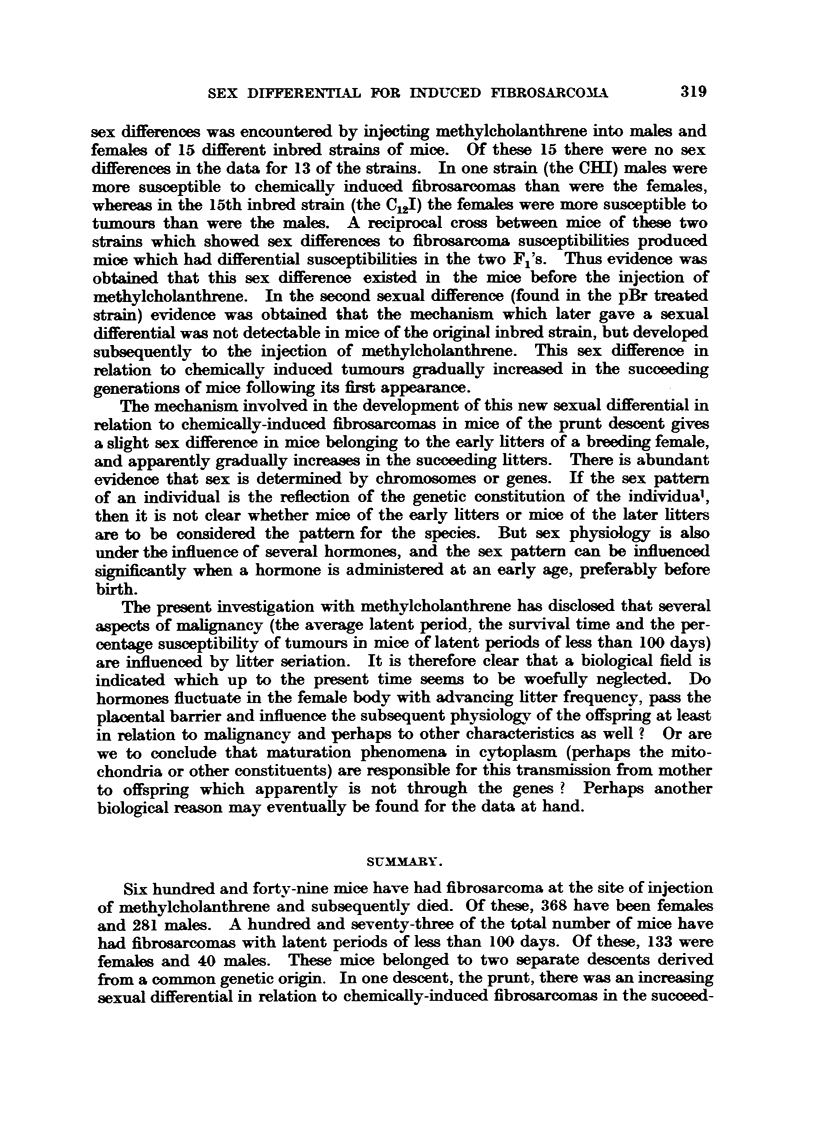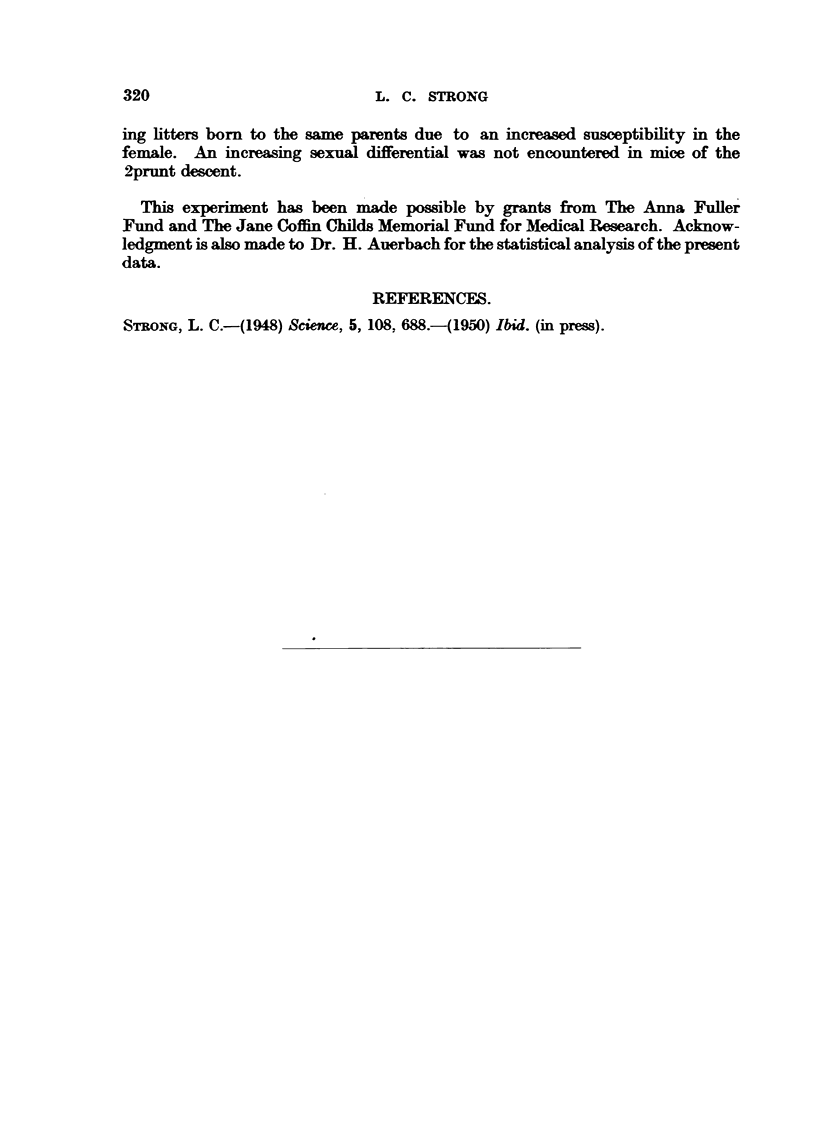# A Sex Differential for Chemically Induced Fibrosarcoma Associated with Litter Seriation

**DOI:** 10.1038/bjc.1950.29

**Published:** 1950-09

**Authors:** L. C. Strong


### A SEX DIFFERENTIAL FOR CHEMICALLY INDUCED FIBROSARCOMA ASSOCIATED WITH LITTER SERIATION.

L. C. STRONG.

*From the Department of Anatomy, Yale University School of Medicine.*

Received for publication May 23, 1950.

DATA have been published which indicate that the average latent period for all methylcholanthrene-induced fibrosarcomas (Strong, 1948) and the survival time (Strong, 1950) of mice developing such tumours are both influenced by litter seriation. The latent period (time between the subcutaneous injection of the carcinogen and the initiation or the initial growth of the ensuing fibrosarcoma) was variously affected in the different strains by the litter to which the mouse belonged. In mice of some strains (prunt and $F_1$ of $C_{67} \times$ Brs) the latent period (Strong, 1948) for the appearance of fibrosarcomas decreased in the successive litters of the same pair of mice, while in other strains susceptibility to fibrosarcomas was relatively constant. Unpublished data have also disclosed that in one subline of mice the average latent period of fibrosarcomas increases in mice of the successive litters. Thus a variable mechanism is indicated in the different sublines which influences susceptibility to fibrosarcomas, and this mechanism is associated with litter seriation. The survival time (Strong, 1950) of mice growing a chemically-induced fibrosarcoma (time between the initial growth of the malignancy and the death of the individual) is also affected by litter seriation. When a mouse developed a fibrosarcoma in less than 100 days following the subcutaneous injection of methylcholanthrene it survived an average of 52 days with the progressive growth of the tumour if it belonged to a first litter. In the succeeding litters mice of the same age and latent period survived longer and longer with growing fibrosarcomas until, if they belonged to an eighth litter, they lived on an average of 130 days.

In this paper data on the average percentage incidence of methylcholanthrene-induced fibrosarcomas are given for mice which develop their malignancy in less than 100 days. Data on the sexes of the mice are separated.

The present observations have been made on two sublines of the pBr strain of mice. The origin of these sublines will only be discussed briefly here. The pBr subline was separated from the original NHO descent in the $F_{14}$ generation of sib inbreeding. The mice are marked by the genes for pink eye, brown and non agouti (genetic constitution ppbbaa). These genes were derived from mice of the ancestral stocks JK and N which were used in the origin of the NHO strain. The first three generations (CBAN $\times$ JK) were used for breeding purposes only. From the $F_4$ generation onward for several generations both parents were injected with 1 mg. of methylcholanthrene at 60 days of age. In the $F_{17}$ generation of continued brother-to-sister matings four mice (born 1.xi.45) were set aside from the experimental animals receiving methylcholanthrene, and have been continued as an untreated descent ever since. These mice are now in the $F_{30}$ generation. This untreated descent has been given the symbol prunt. At the time of the separation of prunt from pBr descent, the average latent period for the appearance of methylcholanthrene-induced fibrosarcomas of the direct maternal ancestry was 369·5 days. In the $F_{20}$ generation of the experimental pBr descent receiving methylcholanthrene, a single pair of mice was set aside and continued as a second untreated descent. This second group received the symbol 2prunt. At the time of separation the average latent period for methylcholanthrene-induced fibrosarcomas of the direct maternal ancestry was 393·0 days. The original mice of the 2prunt descent were born 14.vii.47. At present this 2prunt descent is in the $F_{28}$ generation of inbreeding. These two untreated descents, prunt and 2prunt, should possess genetic similarity, and any biological differences that they have may be due either to divergent segregation from a residuum of genetic heterozygosity (probably very small after 17 generations of brother-to-sister matings) or to new biological variability which has appeared in one or both untreated descents following their separation from a common ancestry.

At 60 days of age the offspring of the untreated prunt and 2prunt sublines were injected subcutaneously with 1·0 mg. of methylcholanthrene dissolved in 0·1 c.c. of sesame oil. All mice were kept in an air-conditioned laboratory with temperature controlled between 70 and 73° F. and the humidity between 50 and 60 per cent. The mice were fed a standard diet of Nurishmix pellets (Pratt Food Company) and water *ad libitum*. A supplement of mixed grains (oats, wheat and sunflower seeds) and calf meal pellets were given to the mice once a week. Enriched Bond bread soaked in milk and Squibb's cod-liver oil was also given once a week. The mice were examined periodically for tumours, and the latent period taken to be the time at which a *firm* nodule at the site of the injection of the carcinogen began to increase progressively in size. Mice which developed tumours at sites other than the one at which the carcinogen had been injected were tabulated separately. A determination of histological types of tumours was obtained by saving tissues from the tumour at the time of the autopsy of the mouse.

Table I gives the percentage incidence of tumours in mice which developed in less than 100 days (Column *e*), together with the data obtained on all tumours at the site of injection of the carcinogen irrespective of latent period (Column *d*). Data on the total number of mice injected with methylcholanthrene (Column *a*), the number of mice which died without developing any tumour (Column *b*), and the total number of mice showing tumours irrespective of latent period and site are also given (Column *c*). Since the two separate descents disclose similar genetic origin and show similar trends by which the females are consistently more susceptible to fibrosarcomas than the males, the data for both series may be added together. However, the present analysis of trends of susceptibility in successive litters discloses differences, and therefore the two strains, the prunt and the 2prunt, should be kept distinct. The data contained in Table I for the prunt descent, Column *e*/Column *a*, are given graphically in Fig. 1.

Up to the present time 477 mice of the prunt descent have been injected with methylcholanthrene. Of these, 250 were females and 227 were males : 337 have developed fibrosarcomas at the site of the injection and have died. Of this total,

TABLE I.—*Incidence of Tumours.*

| Stock. | Litter. | *a* Total injected. ♀ | *a* Total injected. ♂ | *b* Died, no tumour. ♀ | *b* Died, no tumour. ♂ | *c* Mice + tumours. ♀ | *c* Mice + tumours. ♂ | *d* Local tumours. ♀ | *d* Local tumours. ♂ | *e* Local tumours <100 days. ♀ | *e* Local tumours <100 days. ♂ | %. e/a. ♀ | %. e/a. ♂ | %. e/d. ♀ | %. e/d. ♂ |
|---|---|---|---|---|---|---|---|---|---|---|---|---|---|---|---|
| prunt | 1 | 26 | 35 | 1 | 3 | 25 | 32 | 18 | 18 | 4 | 5 | 15·4 | 14·3 | 22·2 | 27·8 |
| | 2 | 38 | 40 | 3 | 2 | 35 | 38 | 32 | 22 | 8 | 3 | 21·1 | 7·5 | 25·0 | 13·6 |
| | 3 | 39 | 44 | 4 | 6 | 35 | 38 | 33 | 33 | 8 | 3 | 20·5 | 6·8 | 24·2 | 9·1 |
| | 4 | 58 | 43 | 4 | 5 | 54 | 38 | 44 | 29 | 17 | 3 | 29·3 | 7·0 | 38·6 | 10·3 |
| | 5 | 39 | 37 | 1 | 6 | 38 | 31 | 28 | 25 | 12 | 6 | 30·8 | 16·2 | 42·9 | 24·0 |
| | 6 | 31 | 14 | 0 | 1 | 31 | 13 | 21 | 11 | 11 | 1 | 35·5 | 7·1 | 52·4 | 9·1 |
| | 7 | 14 | 12 | 0 | 0 | 14 | 12 | 12 | 8 | 7 | 0 | 50·0 | 0·0 | 58·3 | 0·0 |
| | 8 | 5 | 2 | 0 | 0 | 5 | 2 | 2 | 1 | 1 | 0 | 20·0 | 0·0 | 50·0 | 0·0 |
| 2prunt | 1 | 33 | 26 | 0 | 2 | 33 | 24 | 24 | 16 | 6 | 3 | 18·2 | 11·5 | 25·0 | 18·8 |
| | 2 | 34 | 25 | 1 | 3 | 33 | 22 | 24 | 15 | 13 | 2 | 38·2 | 8·0 | 54·2 | 13·3 |
| | 3 | 62 | 48 | 2 | 4 | 60 | 44 | 37 | 29 | 8 | 4 | 12·9 | 8·3 | 21·6 | 13·8 |
| | 4 | 60 | 42 | 0 | 2 | 60 | 40 | 36 | 27 | 15 | 3 | 25·0 | 7·1 | 41·7 | 11·1 |
| | 5 | 51 | 51 | 0 | 5 | 51 | 46 | 32 | 30 | 12 | 5 | 23·5 | 9·8 | 37·5 | 16·7 |
| | 6 | 14 | 14 | 2 | 1 | 12 | 13 | 5 | 6 | 2 | 2 | 14·3 | 14·3 | 40·0 | 33·3 |
| | 7 | 18 | 15 | 2 | 1 | 16 | 14 | 10 | 5 | 5 | 0 | 27·8 | 0·0 | 50·0 | 0·0 |
| | 8 | 16 | 10 | 1 | 3 | 15 | 7 | 10 | 6 | 4 | 0 | 25·0 | 0·0 | 40·0 | 0·0 |

190 were females and 147 were males. Eighty-nine mice have developed fibrosarcomas at the site of injection with latent periods <100 days and have died. Of these, 68 were females and 21 were males. Thus 27·2 per cent of the females developed fibrosarcomas within less than 100 days of latent period, whereas only 9·2 per cent of the males developed similar tumours during the same period, a difference of 18·0 per cent in tumour susceptibility.

FIG. 1.—Litter seriation is plotted on the base line and percentage incidence of fibrosarcomas with latent periods less than 100 days on the vertical line. Data for females on the solid line and data for males on the short dash line. Straight lines determined by the system of least squares are drawn through the points for successive litters.

In the statistical analysis of the prunt results straight line trends were developed from the observed data by the method of least squares (Fig. 1). Then, the equations for these trends were computed. The trend for the percentage incidence of tumours developing in less than 100 days among females of successive litters was found to be $Y = 8{\cdot}5 + 5{\cdot}1x$, with a standard deviation of the slope of $\pm 0{\cdot}741$. Thus, with each successive litter the tumour incidence among females increased by $5{\cdot}1 \pm 0{\cdot}741$ per cent. Comparison of this value with a no-trend or 0-slope value by *t* test reveals that the observed trend is statistically significant (since $P = <0{\cdot}01$).

Similarly the trend for the percentage incidence of tumours developing in less than 100 days among males of successive litters was found to be $Y = 13{\cdot}2 - 1{\cdot}2x$, with a standard deviation of the slope of $\pm 0{\cdot}968$. Thus, with each successive litter the tumour incidence among males decreased by $1{\cdot}2 \pm 0{\cdot}968$ per cent. This trend is not significant (since $P = >0{\cdot}05$).

Therefore the conclusion is warranted that in the prunt descent there is a progressively increasing sex differential in tumour development in response to methylcholanthrene in mice of successive litters. This sex differential results from a statistically significant increasing susceptibility of the females in successive litters, while the male susceptibility under the same circumstances shows no significant change.

A similar analysis of the data for the 2prunt descent shows no significant increasing or decreasing sexual differential in the succeeding litters.

The analysis of the data thus discloses that there is an increasing sex differential in relation to chemically induced fibrosarcomas in successive litters in mice of the prunt descent. This is due to an increased susceptibility in the female. This however apparently is not the case in mice of the 2prunt descent. Both descents have had a common ancestry for 17 brother-to-sister matings. Thus the two descents should be considered as belonging to the *same* inbred strain of mice. The biological difference between mice of the two descents as indicated in this paper may therefore not be genetic. It is known that methylcholanthrene changes cancer susceptibility. Mice of the two untreated pBr descents (prunt and 2prunt) used in this investigation were freed from the injection of methylcholanthrene for at least 5 generations. It does not seem possible, however, that the effects of methylcholanthrene could be transmitted through 5 generations without having a change in genetic constitution. The difference between the mice of the two descents must, therefore, be associated with a mechanism which changes in litter seriation at least in some strains of mice. In the 2prunt descent the maximal sexual differential is found in mice of the second litters (30·2 per cent), and this sex differential fluctuates in the succeeding litters and thus shows no significant trend. However, the data of the 2prunt descent are complicated by a high value for females of the second litter (38·2 per cent) and a high value for males of the sixth litter (14·3 per cent). Further data may alter the present interpretation for the 2prunt descent.

### DISCUSSION.

Rarely has a sex difference on chemically induced fibrosarcomas been observed. During the past ten years with the use of large numbers of mice injected with methylcholanthrene, only two previous cases have been observed. One of these sex differences was encountered by injecting methylcholanthrene into males and females of 15 different inbred strains of mice. Of these 15 there were no sex differences in the data for 13 of the strains. In one strain (the CHI) males were more susceptible to chemically induced fibrosarcomas than were the females, whereas in the 15th inbred strain (the $C_{12}I$) the females were more susceptible to tumours than were the males. A reciprocal cross between mice of these two strains which showed sex differences to fibrosarcoma susceptibilities produced mice which had differential susceptibilities in the two $F_1$'s. Thus evidence was obtained that this sex difference existed in the mice before the injection of methylcholanthrene. In the second sexual difference (found in the pBr treated strain) evidence was obtained that the mechanism which later gave a sexual differential was not detectable in mice of the original inbred strain, but developed subsequently to the injection of methylcholanthrene. This sex difference in relation to chemically induced tumours gradually increased in the succeeding generations of mice following its first appearance.

The mechanism involved in the development of this new sexual differential in relation to chemically-induced fibrosarcomas in mice of the prunt descent gives a slight sex difference in mice belonging to the early litters of a breeding female, and apparently gradually increases in the succeeding litters. There is abundant evidence that sex is determined by chromosomes or genes. If the sex pattern of an individual is the reflection of the genetic constitution of the individua', then it is not clear whether mice of the early litters or mice of the later litters are to be considered the pattern for the species. But sex physiology is also under the influence of several hormones, and the sex pattern can be influenced significantly when a hormone is administered at an early age, preferably before birth.

The present investigation with methylcholanthrene has disclosed that several aspects of malignancy (the average latent period, the survival time and the percentage susceptibility of tumours in mice of latent periods of less than 100 days) are influenced by litter seriation. It is therefore clear that a biological field is indicated which up to the present time seems to be woefully neglected. Do hormones fluctuate in the female body with advancing litter frequency, pass the placental barrier and influence the subsequent physiology of the offspring at least in relation to malignancy and perhaps to other characteristics as well ? Or are we to conclude that maturation phenomena in cytoplasm (perhaps the mitochondria or other constituents) are responsible for this transmission from mother to offspring which apparently is not through the genes ? Perhaps another biological reason may eventually be found for the data at hand.

### SUMMARY.

Six hundred and forty-nine mice have had fibrosarcoma at the site of injection of methylcholanthrene and subsequently died. Of these, 368 have been females and 281 males. A hundred and seventy-three of the total number of mice have had fibrosarcomas with latent periods of less than 100 days. Of these, 133 were females and 40 males. These mice belonged to two separate descents derived from a common genetic origin. In one descent, the prunt, there was an increasing sexual differential in relation to chemically-induced fibrosarcomas in the succeeding litters born to the same parents due to an increased susceptibility in the female. An increasing sexual differential was not encountered in mice of the 2prunt descent.

This experiment has been made possible by grants from The Anna Fuller Fund and The Jane Coffin Childs Memorial Fund for Medical Research. Acknowledgment is also made to Dr. H. Auerbach for the statistical analysis of the present data.

### REFERENCES.

STRONG, L. C.—(1948) *Science*, **5**, 108, 688.—(1950) *Ibid.* (in press).